# Energy Expenditure and Enjoyment During Active Video Gaming Using an Adapted Wii Fit Balance Board in Adults with Physical Disabilities: Observational Study

**DOI:** 10.2196/11326

**Published:** 2019-02-01

**Authors:** Laurie A Malone, Mohanraj Thirumalai, Sangeetha Padalabalanarayanan, Whitney N Neal, Sean Bowman, Tapan Mehta

**Affiliations:** 1 University of Alabama at Birmingham/Lakeshore Foundation Research Collaborative Birmingham, AL United States

**Keywords:** exergaming, video games, exercise, physical activity, disability, energy expenditure, enjoyment

## Abstract

**Background:**

Individuals with physical disabilities have fewer opportunities to participate in enjoyable physical activity. One option for increasing physical activity is playing active video games (AVGs); however, many AVGs are inaccessible or offer limited play options.

**Objective:**

This study aimed to examine energy expenditure and enjoyment in adults with mobility impairment during AVG play using off-the-shelf (OTS) and adapted versions of the Wii Fit balance board (Nintendo).

**Methods:**

During visit 1, participants completed a functional assessment and the familiarization period. During visit 2, metabolic data were collected during a 20-minute baseline and four 10-minute bouts of Wii Fit Plus game play, with two bouts on each of the boards. During the resting period, participants completed the Physical Activity Enjoyment Scale (PACES). Statistical analyses were computed using SPSS software. Data were analyzed separately for individuals who were able to play while standing on both boards (StdStd); those who could not play while standing on the OTS board, but were able to play while standing on the adapted board (aStd); and those who could only play while sitting on the adapted board (aSit).

**Results:**

Data were collected for 58 participants (StdStd, n=17; aStd, n=10; aSit, n=31). The sample included 31 men and 27 women with a mean age of 41.21 (SD 12.70) years. Energy expenditure (metabolic equivalent [MET]) during game play was significantly greater than that during rest for all players. Only 17 participants (StdStd group) were able to play using the OTS board. During game play on the adapted board, the average MET values for the two game sets were 2.261 (SD 0.718) kcal/kg/hour and 2.233 (SD 0.751) kcal/kg/hour for the aSit group, 3.151 (SD 1.034) and 2.990 (SD 1.121) for the aStd group, and 2.732 (SD 0.655) and 2.777 (SD 0.803) for the StdStd group. For game play on the adapted board, self-reported ratings of perceived exertion on a 0-10 scale suggested greater exercise intensity levels, with median scores ranging from moderate (3) to very hard (7). The PACES scores indicated that all players enjoyed using the adapted board, with a median score of 4 on a 5-point scale.

**Conclusions:**

The adapted Wii Fit balance board provided an opportunity for individuals with mobility impairments, including wheelchair users, to engage in AVG. All participants were able to utilize the adapted controller and enjoyed the AVG activity. Although the average MET values achieved during AVG represented light-intensity exercise (<3 METs), 16% of sitting participants and 41% of standing participants achieved moderate-intensity exercise (3-6 METs) in at least one of the games. Factors not accounted for, which may have influenced the intensity of exercise, include game selection, limited familiarization period, and discomfort wearing the COSMED portable metabolic system for measurement of oxygen consumption. Accessible AVG controllers offer an innovative approach to overcome various barriers to participation in physical activity. The next steps include assessment of an AVG intervention using an adapted board gaming controller on health and fitness outcomes.

**Trial Registration:**

ClinicalTrials.gov NCT02994199; https://clinicaltrials.gov/ct2/show/NCT02994199 (Archived by Webcite at http://www.webcitation.org/75fc0mN39).

## Introduction

Physical inactivity is significantly higher in people with disabilities due to fewer opportunities and countless barriers to engaging in leisure-time physical activity [1-7]. Lack of transportation, inaccessible fitness facilities, absence of staff trained to work with people with disabilities, and boredom associated with the use of standard exercise equipment contribute to higher sedentary behaviors in this population [1-7]. Replacing sedentary behaviors with active video games (AVGs) holds promise as a way to reduce those barriers and increase leisure-time physical activity in people with disabilities [8-12]. Moreover, AVGs have the potential to be a “gateway experience” to physical activity, suggesting that such games may open the door to interest and participation in other forms of physical activity for persons with disabilities [10].

AVGs, also called “exergames,” are video games that require actions of large body parts (like the trunk or upper or lower extremity) or the whole body to control game play as opposed to games that only require hand or finger movements to play. Gaming systems that have AVG capability include the Nintendo Wii, Sony PlayStation Move, and Microsoft Xbox Kinect. Depending on the design of games and control systems used, AVGs have been shown to be an enjoyable leisure-time physical activity option to replace sedentary screen time [13-17], with the potential to increase cardiorespiratory fitness and enhance balance and functional mobility [15,16,18-28]. Furthermore, AVGs show promise as an enjoyable physical activity alternative for individuals with disabilities, largely because they overcome certain common barriers to physical activity such as transportation and access to adequate facilities [10,11].

It is important to determine whether the level of physical activity offered by AVGs is high enough to achieve the same fitness and health benefits offered by traditional exercise. Several studies have reported that increases in energy expenditure are sufficient for maintaining and improving health in individuals with mobility impairments such as cerebral palsy [12,29-32], spinal cord injury [33,34], and stroke [29,35-37]. A literature review by Deutsch and colleagues [29] sought to determine whether evidence existed to support the use of video games for the promotion of fitness and wellness for adults after stroke (moderate severity) and those with cerebral palsy (mild severity). It was found that both groups were able to achieve moderate energy expenditure by playing Nintendo Wii, Sony Playstation, and Xbox Kinect games. In another study by Hurkmans et al [31], adults with cerebral palsy who were able to stand without support were asked to play Wii Sports tennis and boxing while their energy expenditure was measured using a portable gas analyzer. The results of the study showed that participants achieved moderate-intensity exercise during both games. A similar study by Robert et al [32] evaluated exercise intensity in children with cerebral palsy who were able to stand and children without cerebral palsy. They found similar exercise intensity levels for both groups, suggesting that children with cerebral palsy who are able to play video games while standing can obtain exercise-related benefits that are similar to those obtained by children without cerebral palsy. A systematic review by Mat Rosly et al [33] found that adults with spinal cord injury were able to achieve the recommended moderate-to-vigorous physical activity levels proposed by the American College of Sports Medicine. A case study of two young adults showed that persons with spinal cord injury are able to achieve moderate intensity–level heart rates when playing boxing on the Nintendo Wii while seated [34]. Furthermore, when compared with conventional boxing, Mat Rosly et al [38] found that individuals with spinal cord injury were able to achieve the same moderate-intensity level as that when playing exergame boxing. Another study determined showed poststroke, individuals were able to play Wii tennis and Wii boxing at a moderate-intensity level (classified using the American College of Sports Medicine and the American Heart Association guidelines as 3-6 metabolic equivalents [METs]) [35]. Another study compared the performance of playing Nintendo Wii and Sony Xbox 360 in adults poststroke and individuals without a disability [36]; the study found that AVG game play while standing produced moderate-intensity exercise levels and poststroke participants approached anaerobic metabolism when playing in the sitting position.

Although AVGs hold promise as an opportunity to increase physical activity in people with disabilities, physical limitations such as decreased motor control, range of motion, muscle strength, ambulatory status, and balance limit AVG accessibility for a large portion of this population [10,12,39]. For example, the majority of the studies noted above for individuals with cerebral palsy and poststroke comprised individuals with mild-to-moderate mobility impairments who were able to play the AVGs without adapted equipment. Similarly, limited AVG play options are available for people who are unable to stand, have balance problems or poor motor control, or cannot use their lower body to perform game movements [10]. For instance, floor-pad game controllers used by AVGs (eg, Dance, Dance Revolution and Wii Outdoor Challenge) have obvious accessibility limitations. Although motion-controlled AVGs offer slightly greater access, accelerometer-based hand controllers like those used by the Sony PlayStation Move and Nintendo Wii platforms often require rapid and precise movements for successful play, and AVGs using the camera-based controller of Microsoft Kinect typically require the player to be standing for proper game function. Therefore, AVG adaptations to game controllers are essential in order to offer people with disabilities options for AVGs for moderate-to-vigorous exercise in environments such as their home and communities [11]. Offering adapted controllers for AVG play to people with disabilities is also a necessary first step toward examining the feasibility of such controllers for increasing energy expenditure.

The development of adapted game hardware not only offers an innovative approach to overcoming numerous barriers to exercise in people with disabilities, but also provides an enjoyable form of exercise to this population. Successful adaptations to game controllers and interfaces that allow people with disabilities to play video games have been developed [40-43]; however, there has been limited research and development efforts focused on improving the accessibility of commercially available gaming controllers for use with AVGs. The Rehabilitation Engineering Research Center on Interactive Exercise Technologies and Exercise Physiology for People with Disabilities at the University of Alabama at Birmingham/ Lakeshore Foundation Research Collaborative examined the accessibility of video game controllers including the Wii Fit balance board system. Data on game play, participants’ ability to use the controllers, user feedback, and research staff qualitative observations indicated that the Wii balance board was in need of adaptation for successful game play. These data were fed to the engineering team for development of an adapted gaming balance board.

**Figure 1:**
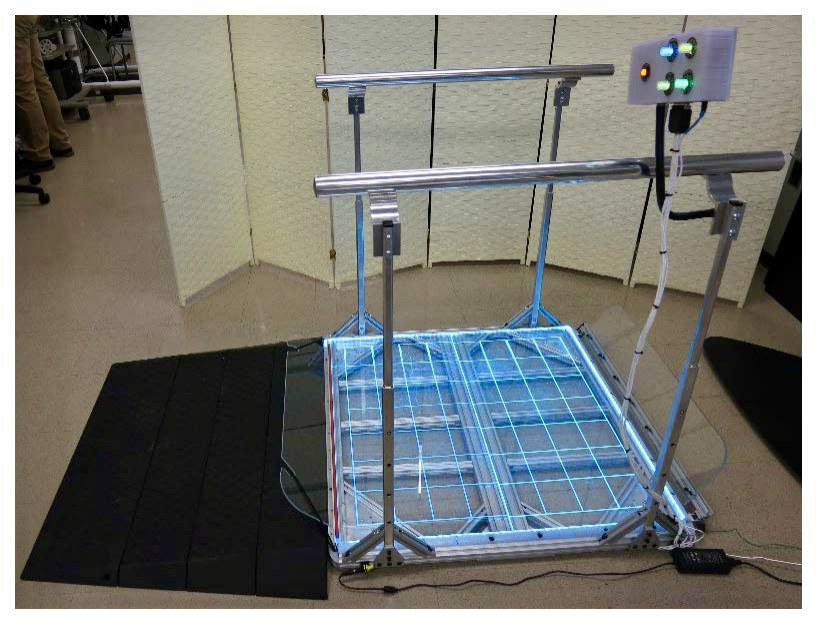
Adapted Wii Fit balance board with a ramp for wheelchair access, adjustable-height handrails, and control box. Usable space on the adapted balance board measures 91.5 cm x 91.5 cm, with the load cells placed at each of the four corners. The electrical components of the off-the-shelf Wii Fit balance board were reconfigured and integrated into the new form factor and electrical design of the adapted board.

The primary deficiencies in the balance board included a small platform area (19.5”×12”), the inability to use a stabilization assistive device (ie, walker or cane), and the requirement of a full range of motion for responsive game play. To address these deficiencies and increase accessibility, the balance board was redesigned to feature a much larger platform area (40”×38”), built-in lateral stabilization supports (ie, handrails), and adjustable sensitivity for center of balance support [44]. The adaptations were selected to not only enable wheelchair users to use the balance board, but to make it a universal device with enhanced safety for all users (Figure 1).

Usability of the off-the-shelf (OTS) and adapted balance board controllers was evaluated in individuals with mobility impairments using the System Usability Scale (SUS) [44]. The user-centered design approach resulted in an adapted version of the Wii Fit balance board, which met the needs of a variety of users. Results demonstrated a successful adaptation and increase in usability of the adapted balance board, with the adapted board scoring significantly greater mean SUS scores than those of the off-the-shelf board.

Despite the fact that a variety of successful adaptations to AVG game controllers and interfaces that allow people with disabilities to play AVGs have been developed by rehabilitation engineers and assistive technology specialists, there have been limited research and development efforts focused on measuring energy expenditure. Modifications of these game controllers must be successful in allowing people with disabilities to not only play AVGs, but also achieve levels of energy expenditure that are similar to the levels of people without disabilities.

The purpose of this study was to examine energy expenditure and enjoyment in adults with physical disabilities, specifically those with mobility impairments (ie, inability to stand, balance issues, poor motor control, and inability to use lower extremity for game play), during AVG play using OTS and adapted versions of the Wii Fit balance board.

## Methods

### Design and Setting

The study was conducted in the Exercise and Sport Science Laboratory at Lakeshore Foundation (Birmingham, AL), a community organization that provides physical activity, sport, and recreation opportunities to individuals with physical disability and chronic health conditions (trial registration: ClinicalTrials.gov NCT02994199). For the purposes of this study, participants generally visited the laboratory a total of 3 times within a 3-week period.

### Participants

Eligibility criteria included age ≥18 years, a confirmed diagnosis of lower extremity-mobility limitation (eg, spina bifida, cerebral palsy, muscular dystrophy, 1 year after spinal cord injury, multiple sclerosis, stroke, or limb loss) with partial or full use of upper extremities and use of an assistive device (eg, cane, walker, or wheelchair) or problems with gait, balance, or coordination. Participants were excluded if they had an unstable cardiovascular condition, a visual impairment that interfered with playing video games, or weight over 350 lbs (159 kg) including their assistive device.

### Procedures

#### Visit 1

During the first visit, informed consent/assent was obtained, and demographic and health history information was documented. An assessment of each participant’s functional ability was conducted as described below. In addition, participants were familiarized with the equipment (K4b2 portable metabolic system, COSMED, Rome, Italy) used for the study and the video games that would be played during subsequent visits. Participants played a portion of or the entire game for all games that were to be used during testing.

For the assessment of physical function during the first visit, each participant performed 18 functional movement tasks from the International Classification of Functioning, Disability and Health (ICF) [45,46]. Participants completed each task individually and were scored according to their difficulty in completing the task on a scale of 0 to 4. As defined in the ICF manual, the scoring was as follows: 0, “No difficulty”; 1, “Mild difficulty”; 2, “Moderate difficulty”; 3, “Severe difficulty”; and 4, “Complete difficulty.” The specific ICF tasks selected for use in this study were based on a consensus among the research staff about which mobility activities listed in the ICF would potentially be required for AVG play (eg, standing, reaching, throwing, and jumping) based on observations during the pilot testing. Scores on each of the 18 tasks were added together as a composite to represent participant physical function [9]. A lower composite score indicated greater functional ability on the selected tasks.

In addition to the functional assessment, participants also completed a series of questions from the HealthMeasures resources [47], which were used as an assessment of the individual’s own perspective regarding their functional ability. Questions were taken from the Patient Reported Outcomes Measurement Information System (PROMIS) [48]. The series comprised questions from PROMIS SFv1.0 Physical Function 20a and PROMIS SFv1.0 Physical Function Samples with Mobility Aid. Questions addressed issues of how difficult a variety of daily tasks (ie, vacuuming, yard work, walking, and bathing) were to complete (5 point scale, “without any difficulty” to “unable to do”; 14 questions), whether their health limited their ability to complete certain activities (ie, carry groceries, strenuous sports, walk a mile; 5 point scale, “not at all” to “cannot do”; 6 questions), and their ability to stand and move with and without support (“yes” or “no,” 1 question; 5 point scale, “without any difficulty” to “unable to do”, 10 questions).

#### Active Video Game Play

The second visit consisted of exercise testing during video game play. Upon arrival, participants were set up with the COSMED K4B2 portable metabolic system and a Polar (Kempele, Finland) heart rate monitor to assess pulmonary gas exchange and indirect calorimetry. Data collection began with a 20-minute rest period to measure the resting energy expenditure. For the rest period, participants sat quietly with no speaking or distractions besides light reading of a magazine or viewing their cellular phone. Next, game play began with continued gas exchange and heart rate data collection.

The Nintendo Wii video game console and a video game CD for the Wii Balance Board (Wii Fit Plus, Nintendo) were used for game play. Two game sets were created as outlined in Table 1. Game Set A included Rhythm Kung Fu, Rhythm Parade, Obstacle Course, and Bird’s Eye Bull’s Eye. Game Set B included Island Cycling, Penguin Slide, Hula Hoop, and Ski Slalom. Selected games were chosen in an effort to provide moderate-level physical activity during game play. Games represented popular genres (eg, fitness, sport, and adventure) and had an Entertainment Software Rating Board score of "E"" (everyone).

Participants first played the two game sets on the OTS gaming board and then played the games on the adapted gaming board. The order of the game sets (Set A and Set B) played was randomly assigned to the participant. Each game set was played for 10 minutes with a rest period of 5 minutes afterward [9].

**Table 1:** Description of each game played using the off-the-shelf and adapted balance board.

Mini game		Description
**Wii Fit Plus: Game Set A**		
	Rhythm Kung Fu	The participant follows the Kung Fu movements of Mii characters in time with the rhythm. Movements include left and right punches, two hand punches, and left and right kicks.
	Rhythm Parade	The participant marches in place to the rhythm of the parade while directing the parade with arm movements in coordination with the game.
	Obstacle Course	The participant marches in place to run the character through an obstacle course of swinging balls, moving platforms, and jumps.
	Bird’s-Eye Bull’s-Eye	The participant flaps their arms and moves their body to fly through the course landing on targets spread across the map.
**Wii Fit Plus: Game Set B**		
	Island Cycling	The participant marches in place on the board to pedal the bike while capturing flags that are spread throughout the island.
	Penguin Slide	The participant catches fish by leaning left and right on the balance board to control the Mii.
	Hula Hoop	The participant rotates their trunk/hips in a circular motion on the board to control the Mii hula hooping.
	Ski Slalom	The participant skis down the slope by leaning left and right to control the Mii skier’s course.

At the end of each game set, participants provided a rating of perceived exertion (RPE) on a scale of 0-10, with 0 indicating “Not Tired at All” to 10 indicating “Very, Very Tired.” During the rest periods, participants completed a feedback survey that included the Physical Activity Enjoyment Scale (PACES) [49]. PACES includes 16 statements such as “I enjoyed it,” “It was very exciting,” “I felt bored,” and “It was no fun at all.” All items were rated by the participant on a 5-point scale ranging from 1, “Strongly Disagree” to 5, “Strongly Agree.” After reverse scoring 7 items, a final score was computed by calculating the average of items that were answered (blank items were excluded from the average).

### Data Analysis

Analyses were performed using the Statistical Package for the Social Sciences (IBM Corp, Chicago, IL). Due to the failure of groups to meet normality assumptions as well as a relatively small sample size for some comparisons, we reported the median and interquartile range for all measures and applied the Wilcoxon signed rank nonparametric test to detect significant differences between measures when comparisons were merited [50].

For each of these analyses, participants were divided into one of three unique groups based on their method of game play and level of access: Sitting (aSit), wherein individuals were seated for game play, were unable to access the OTS board, and played only on the adapted board; Standing, wherein individuals used the adapted board only (aStd), as they were able to stand, but due to mobility or balance issues could not access the OTS board, and therefore, they played only on the adapted board; and Standing with both boards (StdStd), wherein individuals were able to stand for game play on both the OTS and adapted boards.

To assess the project aims, a series of analyses were conducted. To test whether energy expenditure during game play was different from the RPE (which could be readily taken for granted but was not assumed), resting METs were compared to game play METs for each subgroup. In general, 1 MET represents the amount of oxygen consumed and the number of calories burned at rest. In addition, for the subgroup StdStd, the change in METs (game play – rest) was compared between the OTS and adapted boards. The intensity of game play was defined as follows: <3 METs, light intensity; 3-6 METs, moderate intensity; >6 METs, vigorous intensity. Heart rate as a measure of exercise intensity was also recorded during all game play sessions. As a subjective rating of energy expenditure, RPE scores were analyzed for both OTS and adapted boards. To examine enjoyment, PACES scores were analyzed for both OTS and adapted boards for participants who were able to play both games; for participants who could only play using the adapted controller, scores were simply reported. Our primary comparison was for the StdStd group in which participants were able play using both the OTS and adapted boards. For the aSit and aStd groups, where participants were unable to play using the OTS board, we chose to report only descriptive measures for the corresponding scores while playing the adapted boards. Furthermore, MET change (game - rest) comparisons between the OTS and adapted board in aSit and aStd groups were not computed as the change score for OTS would be equal to zero.

## Results

A total of 58 participants (aStd, n=10; aSit, n=31; StdStd, n=17) completed two 10-minute bouts of select Wii Fit Plus games on the OTS and adapted boards. The sample included 31 men and 27 women with a mean age of 41.21 (SD 12.70) years. Disabilities included spinal cord injury (n=11), multiple sclerosis (n=9), cerebral palsy (n=8), stroke (n=6), spina bifida (n=5), traumatic brain injury (n=4), limb loss (n=2), transverse myelitis (n=2), and others (n=11).

Heart rate and MET data are reported in Tables 2 and 3. For all conditions, the heart rate was significantly higher during game play than at rest (Table 2). Analysis of the metabolic data indicated that energy expenditure (METs) during game play was significantly greater than resting MET values for all players during both game sets on the adapted and OTS boards (Table 3).

**Figure 2:**
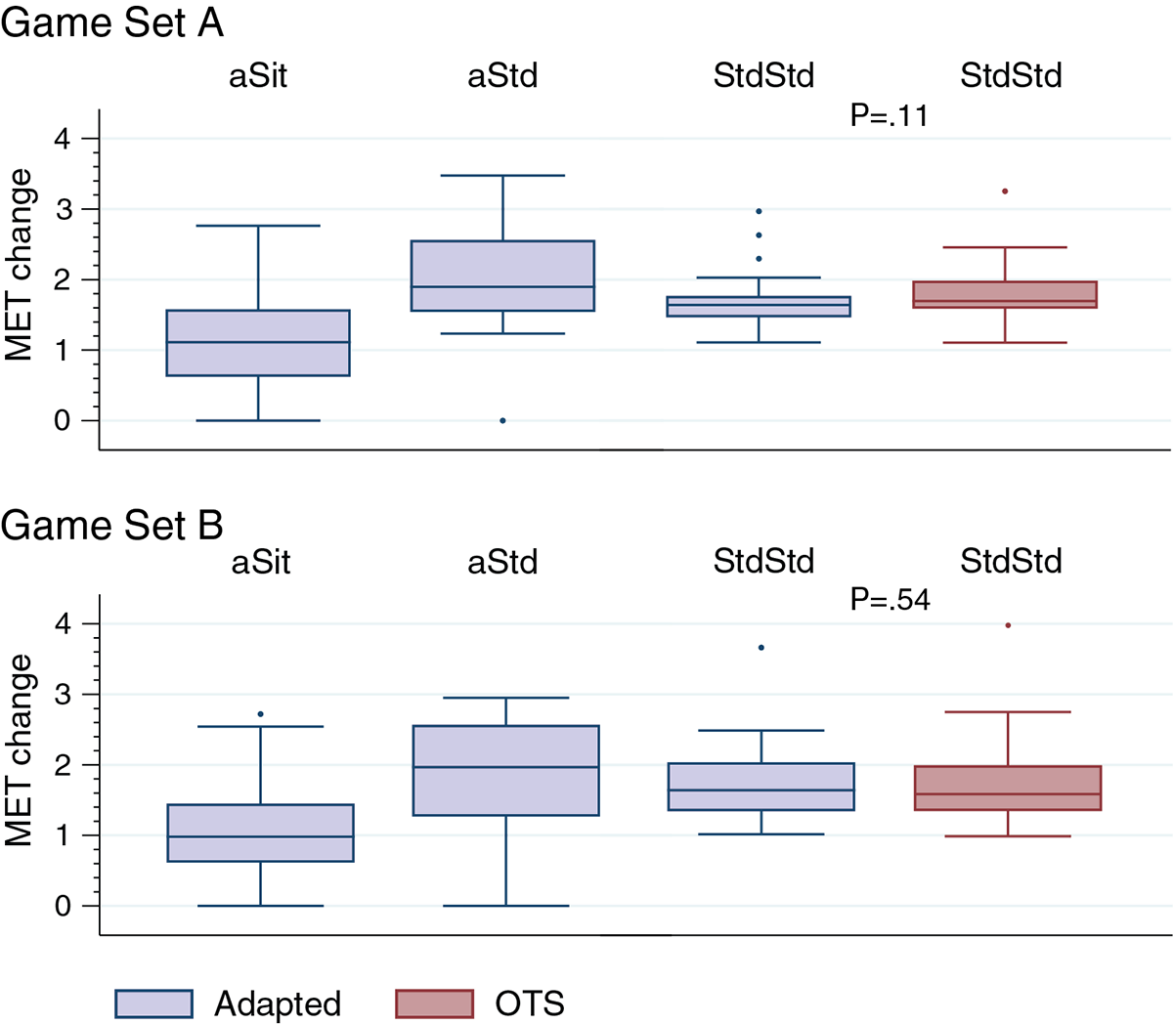
Change in energy expenditure (metabolic equivalents [METs], gameplay – rest) for each group with comparison of MET change between boards (adapted vs off-the-shelf [OTS]) for the StdStd group. Play style groups: aSit - participants who only played seated on the adapted board; aStd - participants who only played standing on the adapted board; StdStd - participants who were able to play standing on both boards. MET-change data for the OTS board are not reported for aSit and aStd groups, as these groups could not play on the OTS board. Computing P values for these comparisons with the assumption of OTS=0 would yield highly statistically significant differences (<.01).

Changes in energy expenditure (game play METs – rest METs) for each group are shown in Figure 2. When comparing MET change values between the OTS and adapted boards for the StdStd group, no significant differences (*P*<.001) were observed. The OTS board data are not reported for aSit and aStd, as these groups could not play on the OTS board and a MET change of zero was assumed for each participant.

**Figure 3:**
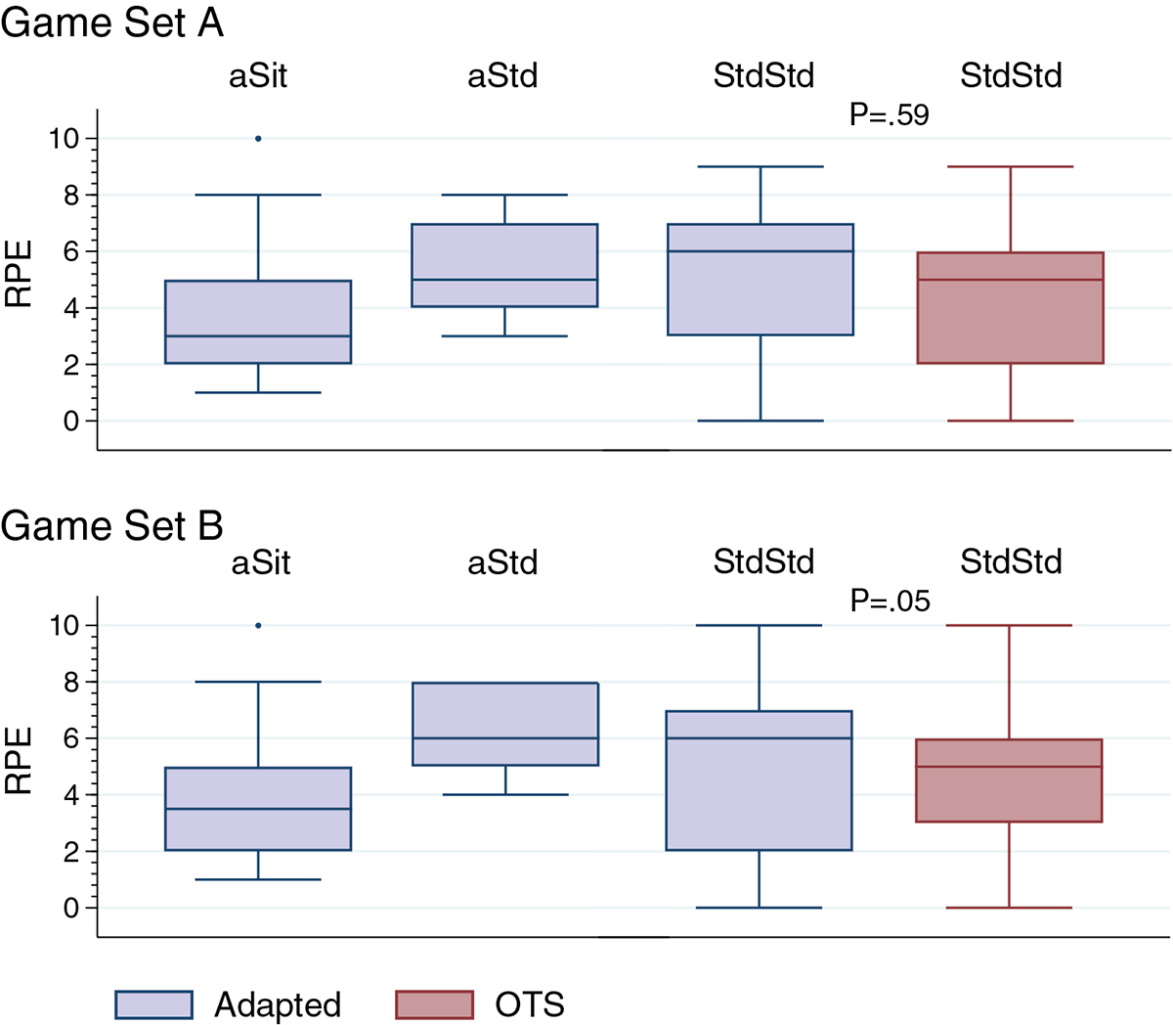
Rating of perceived exertion (RPE) following game play for each group with comparison of RPE between boards(adapted vs off-the-shelf [OTS]) for the StdStd group. Play style groups: aSit - participants who only played seated on the adapted board; aStd - participants who only played standing on the adapted board; StdStd - participants who were able to play standing on both boards.

As a subjective rating of exercise intensity, RPE values were recorded as shown in Figure 3. Median scores are displayed for each of the three groups. For the StdStd group, scores were compared between the two boards. For Set A, RPE did not differ significantly between game play on the OTS and adapted boards. For Set B, RPE values for the adapted board were significantly higher (*P*<.05) than those for the OTS board.

**Figure 4:**
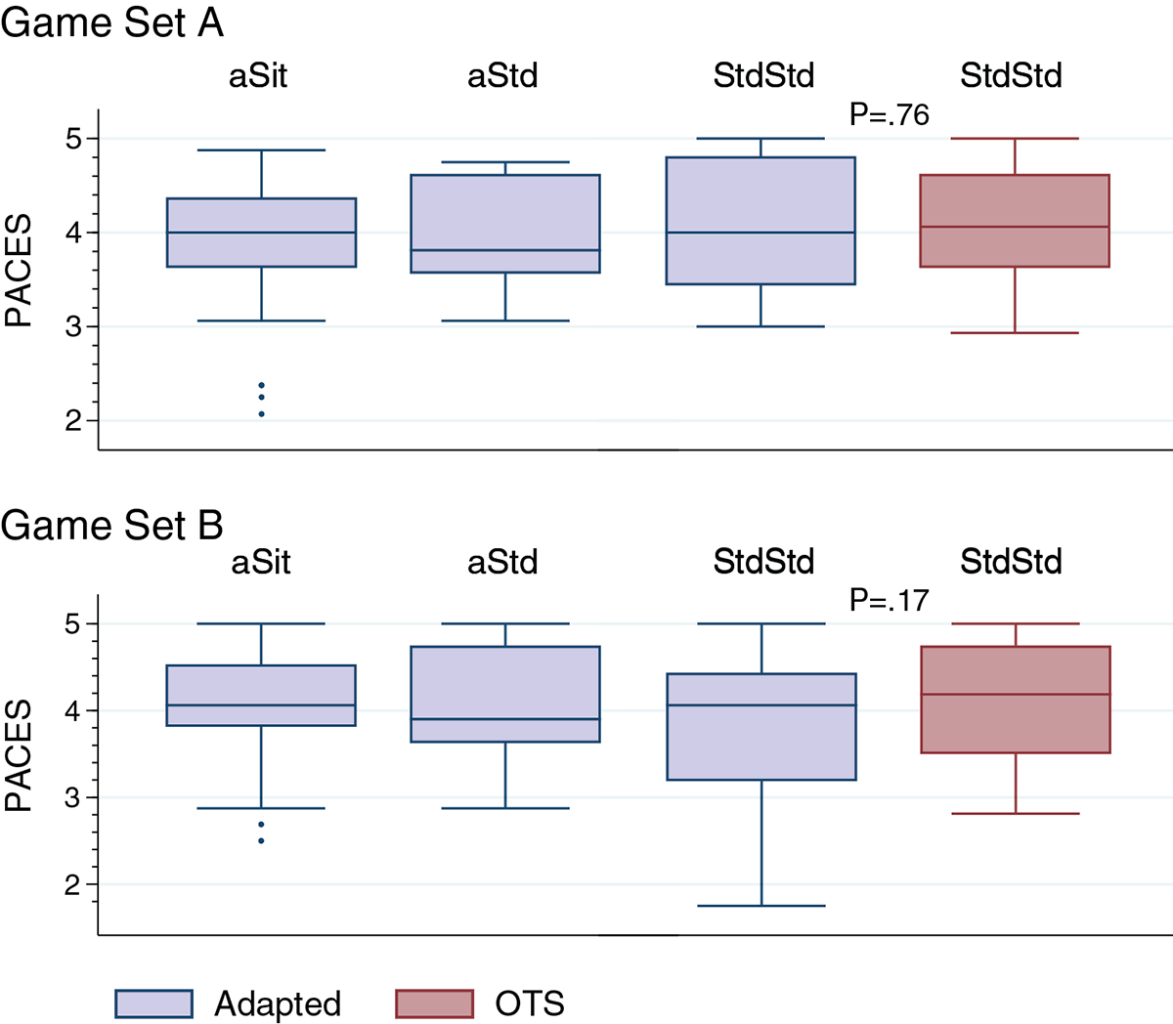
Assessment of game play enjoyment based on Physical Activity Enjoyment Scale (PACES) scores. Play style groups: aSit - participants who only played seated on the adapted board; aStd - participants who only played standing on the adapted board; StdStd - participants who were able to play standing on both boards. PACES data for the off-the-shelf (OTS) board are not reported for aSit and aStd, as these groups could not play on the OTS board.

With regard to enjoyment, there were no significant differences in the PACES scores between the OTS and adapted boards (Figure 4). Median PACES scores for all conditions were 4 or “agree.”

**Table 2:** Resting and game play heart rate on the off-the-shelf and adapted boards.

Play style groups and game set			Game HR^a^, mean (SD)	Game HR, median (IQR^b^)	Resting HR, mean (SD)	Resting HR, median (IQR)	Wilcoxon signed rank *P* value
**Off-the-shelf board**							
	**aSit^c^**				84.288 (12.278)	85.408 (74.985-90.446)	—^d^
		Set A (n=27)	U^e^	U	N/A^f^	N/A	N/A
		Set B (n=26)	U	U	N/A	N/A	N/A
	**aStd^g^**				83.659 (10.861)	86.999 (74.539-90.791)	—
		Set A (n=8)	U	U	N/A	N/A	N/A
		Set B (n=8)	U	U	N/A	N/A	N/A
	**StdStd^h^**				73.118 (11.161)	71.247 (67.099-79.265)	.001
		Set A (n=14)	94.720 (19.783)	86.706 (81.270-106.294)	N/A	N/A	N/A
		Set B (n=14)	96.677 (0.568)	92.436 (78.966-109.271)	N/A	N/A	N/A
**Adapted board**							
	**aSit**				84.288 (12.278)	85.408 (74.985-90.446)	<.001
		Set A (n=27)	95.844 (15.423)	95.108 (84.836-103.678)	N/A	N/A	N/A
		Set B (n=26)	97.798 (15.960)	96.282 (87.430-103.082)	N/A	N/A	N/A
	**aStd**				83.659 (10.861)	86.999 (74.539-90.791)	.01
		Set A (n=8)	109.959 (10.248)	109.547 (102.975-117.275)	N/A	N/A	N/A
		Set B (n=8)	110.250 (13.643)	105.966 (99.863-123.749)	N/A	N/A	N/A
	**StdStd**				73.118 (11.161)	71.247 (67.099-79.265)	.001
		Set A (n=14)	97.074 (21.027)	93.499 (81.296-108.745)	N/A	N/A	N/A
		Set B (n=14)	95.188 (20.475)	89.529 (78.592-109.854)	N/A	N/A	N/A

^a^HR: heart rate.

^b^IQR: interquartile range.

^c^aSit: participants who only played seated on the adapted board.

^d^Not available.

^e^U: Unable to utilize off-the-shelf board for game play.

^f^N/A: not applicable.

^g^aStd: participants who only played standing on the adapted board.

^h^StdStd: participants who were able to play standing on both boards.

**Table 3:** Resting and game play energy expenditure on the off-the-shelf and adapted boards.

Play style groups and game set			Game METs^a^, mean (SD)	Game METs, median (IQR^b^)	Resting METs, mean (SD)	Resting METs, median (IQR)	Wilcoxon signed rank *P* value
**Off-the-shelf board**							
	**aSit^c^**				1.157 (0.375)	1.123 (0.916-1.307)	—^d^
		Set A (n=31)	U^e^	U	N/A^f^	N/A	N/A
		Set B (n=31)	U	U	N/A	N/A	N/A
	**aStd^g^**				1.063 (0.346)	0.902 (0.812-1.269)	—
		Set A (n=10)	—	—	N/A	N/A	N/A
		Set B (n=10)	—	—	N/A	N/A	N/A
	**StdStd^h^**				1.014 (0.235)	1.032 (0.781-1.164)	<.001
		Set A (n=17)	2.839 (0.646)	2.777 (2.623-3.048)	N/A	N/A	N/A
		Set B (n=17)	2.702 (0.568)	2.707 (2.265-2.979)	N/A	N/A	N/A
**Adapted board**							
	**aSit**				1.157 (0.375)	1.123 (0.916-1.307)	<.001
		Set A (n=31)	2.261 (0.718)	2.143 (1.847-2.701)	N/A	N/A	N/A
		Set B (n=31)	2.233 (0.751)	2.203 (1.762-2.567)	N/A	N/A	N/A
	**aStd**				1.063 (0.346)	0.902 (0.812-1.269)	.005
		Set A (n=10)	3.151 (1.034)	2.827 (2.482-3.605)	N/A	N/A	N/A
		Set B (n=10)	2.990 (1.121)	3.028 (2.204-3.675)	N/A	N/A	N/A
	**StdStd**				1.014 (0.235)	1.032 (0.781-1.164)	<.001
		Set A (n=17)	2.732 (0.655)	2.714 (2.443-2.974)	N/A	N/A	N/A
		Set B (n=17)	2.777 (0.803)	2.572 (2.253-3.136)	N/A	N/A	N/A

^a^METs: metabolic equivalents.

^b^IQR: interquartile range.

^c^aSit: participants who only played seated on the adapted board.

^d^Not available.

^e^U: Unable to utilize off-the-shelf board for game play.

^f^N/A: not applicable.

^g^aStd: participants who only played standing on the adapted board.

^h^StdStd: participants who were able to play standing on both boards.

## Discussion

### Overview

Development of AVGs that are accessible to people with disabilities offers an innovative approach to overcoming a number of barriers to participation in physical activity. To date, little effort has been made to design commercially available AVG controllers in a way to promote active play in persons who require a wheelchair for mobility. Although several studies have documented increases in energy expenditure during AVG by individuals with mobility impairments who played standing [30,31,35,36], limited evidence is available regarding outcomes associated with AVG play while sitting. A feasibility study conducted by Rowland and Rimmer [12] modified the parameters for Dance, Dance Revolution game play to allow for arm use by placing the mat on a table and found increases in energy expenditure among three nonambulatory young adults with cerebral palsy. Mat Rosly et al [51] examined game play MET and RPE levels by persons with spinal cord injury utilizing the PlayStation 3 console with two PlayStation Move hand-held controllers. Other attempts have been made to supplement existing exercise equipment with gaming for various populations (ie, spinal cord injury and cerebral palsy) with mobility impairments. Adding game play to wheelchair ergometry makes exercise more enjoyable [52-54], similar to the case with leg cycling [55], while also achieving recommended exercise intensity levels for health-related outcomes.

Recognizing the potential for AVG play to increase energy expenditure among people with more severe mobility impairments led our team to develop an adapted balance board gaming controller [44]. Thus, the objective of the current study was to examine energy expenditure and enjoyment during AVG in persons with mobility impairments when using both OTS and adapted versions of the Wii Fit balance board.

Resting METs were compared to game play METs for each subgroup (based on the degree of mobility impairment) to determine if players actually experienced an increase in energy expenditure during AVG play above the resting values. Given the severity of mobility impairment of some participants, this could not be assumed. The significant differences between game play and resting MET values for all players suggest that AVG play using the adapted controller may provide a leisure-time physical activity option that reduces sedentary time for individuals with a variety of mobility limitations. The increased usability of the gaming board opens the door to AVG play for many people who were previously unable to participate [10,44].

For players who were unable to utilize the OTS board, change in the METs from rest to game play equaled to zero (ie, METs remained at resting values). However, on the adapted board, participants who played while sitting were able to achieve average MET values of 2.261 (SD 0.718) and 2.233 (SD 0.751) kcal/kg/hour on the two game sets each. Average MET values for those who played standing were a bit higher (3.151 [SD 1.034] and 2.990 [SD 1.121] kcal/kg/hour), as expected, given the ability to engage greater muscle mass (ie, lower extremity). Although these average values represent light-to-moderate-intensity exercise, 16% of the seated participants and 41% of the standing participants achieved moderate-intensity exercise on at least one of the games. However, these standard categories based on MET values do not take into account the effect of an individual’s impairment level on intensity of exercise.

In some cases, a self-reported rating of perceived exertion (RPE) on a 0-10 scale suggested greater exercise intensity levels than MET levels recorded, which is similar to the results of an exergaming study in persons with spinal cord injury [51]. Seated players reported a median RPE of 4 (moderate) for both game sets. Players who could only play standing on the adapted board reported somewhat hard-to-moderately hard RPE levels (5-6), while players who played standing on both boards reported moderate-to-somewhat hard RPE levels (4-5).

In a study on healthy young adults, in which participants played various Wii games, exercise intensity varied by the game, ranging from light to moderate [24]. The authors suggested that for games that require controller skill, exercise intensity may be influenced by the player’s prior gaming experience. Furthermore, the benefits of light-intensity exercise are acknowledged even if moderate levels are not reached, which was the case for some participants in our study. As noted in a recent study, replacement of sedentary time with light-intensity physical activity is associated with less mortality in the general population of adults aged ≥40 years [56], with beneficial effects on health outcomes such as blood glucose levels [57].

In addition to the potential for increased energy expenditure, many people perceive AVGs as fun, providing an enjoyable option to perform recommended daily amounts of physical activity. In this study, the PACES scores indicated that all players, sitting and standing, enjoyed playing each of the game sets, with a median score of 4 on a 5-point scale. In a study comparing heavy-bag boxing to AVG boxing in a seated position among persons with spinal cord injury, participants reported more enjoyment during AVG boxing [38]. In another study, game play performance and exercise intensity were positively correlated with AVG enjoyment in youth with mobility impairments [58]. Enjoyment may serve as a determinant of physical activity, further suggesting the need to develop AVG interventions and examine the role of enjoyment on the level of engagement, exercise intensity, and adherence.

### Limitations

This study was not a randomized controlled trial; therefore, no claims can be made regarding causality or efficacy. As an observational study, inherent limitations existed and thereby limit the generalizability of results to the broader community. All participants were recruited from the membership of a community physical activity and recreation center for individuals with physical disabilities. Individuals were, to some degree, physically active with varied AVG experience. Factors that may have influenced intensity of exercise but were not accounted for include game selection, limited familiarization period, and discomfort wearing the COSMED system for oxygen consumption measurement. Although a familiarization period was provided, some degree of game play learning may have occurred during data collection. In addition, given that participants played only a select group of AVGs, potential differences in enjoyment and energy expenditure between OTS and adapted controllers may not have been fully captured. Moreover, the standard categories of exercise intensity based on MET values do not take into consideration the effect of impairment level on exercise intensity. Furthermore, the intrinsic nature of measures examining subjective aspects of exercise such as enjoyment and perceived exertion prevented comparison of these aspects across board type for the participants who were unable to utilize the OTS board. Future studies should expand the participant recruitment pool, examine a broader range of AVGs, provide a more extensive familiarization period, and compare AVG play while utilizing the adapted controllers for other leisure-time physical activities. Further analyses that explore the multivariate correlation and variance-covariance matrices in the outcome measures (RPE, heart rate, MET, PACES) would provide a better understanding of the relationship between exercise intensity and enjoyment and of clinically meaningful differences during AVG in this subpopulation.

### Conclusions

Engagement in physical activity is an important component of a healthy lifestyle and reduces the risk for many serious health conditions such as cancer, heart disease, stroke, and obesity [59]. Individuals with physical disabilities are at an even greater risk for many of these conditions. With limited options for physical activity, finding alternative means in order to stay active is critical for achieving optimal health. The adapted board improved access and allowed participants of all mobility levels to engage in AVG play, removing barriers associated with the OTS board. The adapted game controller provided AVG game play options for individuals unable to stand during play. Players were able to achieve MET values above the resting level, thereby reducing sedentary time. Furthermore, light-to-moderate intensity exercise levels were reached by some participants, providing an enjoyable option for engagement in health-related physical activity.

## Abbreviations

aSit: participants who only played seated on the adapted board
aStd: participants who only played standing on the adapted board
AVG: active video games
HR: heart rate
ICF: International Classification of Functioning, Disability and Health
IQR: interquartile range
MET: metabolic equivalent
OTS: off-the-shelf
PACES: Physical Activity Enjoyment Scale
PROMIS: Patient Reported Outcomes Measurement Information System
RPE: rating of perceived exertion
StdStd: participants who were able to play standing on both boards
SUS: System Usability Scale
